# Hepatitis C testing and re-testing among people attending sexual health services in Australia, and hepatitis C incidence among people with human immunodeficiency virus: analysis of national sentinel surveillance data

**DOI:** 10.1186/s12879-017-2848-0

**Published:** 2017-12-01

**Authors:** David C. Boettiger, Matthew G. Law, Gregory J. Dore, Rebecca Guy, Denton Callander, Basil Donovan, Catherine C. O’Connor, Christopher K. Fairley, Margaret Hellard, Gail Matthews

**Affiliations:** 1 The Kirby Institute, UNSW Sydney, Sydney, NSW Australia; 2 Sydney Sexual Health Centre, Sydney, NSW Australia; 3 The Kirby Institute, UNSW Sydney and Sexual Health Service, Sydney Local Health District, Sydney, NSW Australia; 4 Central Clinical School, Sydney University, Sydney, NSW Australia; 5 Melbourne Sexual Health Centre, Alfred Health and Monash University, Melbourne, Victoria Australia; 6 Viral Hepatitis Elimination Program, Burnet Institute, Melbourne, Victoria Australia

**Keywords:** Hepatitis C virus, Human immunodeficiency virus, Sexual health, Australia

## Abstract

**Background:**

Direct acting antivirals are expected to drastically reduce the burden of hepatitis C virus (HCV) in people living with Human Immunodeficiency Virus (HIV). However, rates of HCV testing, re-testing and incident infection in this group remain uncertain in Australia. We assessed trends in HCV testing, re-testing and incident infection among HIV-positive individuals, and evaluated factors associated with HCV re-testing and incident infection.

**Methods:**

The study population consisted of HIV-positive individuals who visited a sexual health service involved in the Australian Collaboration for Coordinated Enhanced Sentinel Surveillance (ACCESS) between 2007 and 2015. Poisson regression was used to assess trends and to evaluate factors associated with HCV re-testing and incident HCV infection.

**Results:**

There were 9227 HIV-positive individuals included in our testing rate analysis.

Of 3799 HIV-positive/HCV-negative people that attended an ACCESS sexual health service more than once, 2079 (54.7%) were re-tested for HCV and were therefore eligible for our incidence analysis. The rate of HCV testing increased from 17.1 to 51.4 tests per 100 patient years between 2007 and 2015 (*p* for trend <0.01). Over the same period, HCV re-testing rates increased from 23.9 to 79.7 tests per 100 person years (*p* for trend <0.01). A clear increase in testing and re-testing began after 2011. Patients who identified as men who have sex with men and those with a history of injecting drug use experienced high rates of HCV re-testing over the course of the study period. Among those who re-tested, 157 incident HCV infections occurred at a rate of 2.5 events per 100 person years. Between 2007 and 2009, 2010–2011, 2012–2013 and 2014–2015, rates of incident HCV were 0.8, 1.5, 3.9 and 2.7 events per 100 person years, respectively (*p* for trend <0.01). Incident HCV was strongly associated with a history of injecting drug use.

**Conclusions:**

High rates of HCV testing and re-testing among HIV-positive individuals in Australia will assist strategies to achieve HCV elimination through rapid treatment scale up. Continued monitoring of HCV incidence in this population is essential for guiding both HCV prevention and treatment strategies.

**Electronic supplementary material:**

The online version of this article (10.1186/s12879-017-2848-0) contains supplementary material, which is available to authorized users.

## Background

Hepatitis C virus (HCV) is a major contributor to morbidity and mortality in people living with Human Immunodeficiency Virus (HIV) [1–3]. In Australia, it is estimated that around 3000 individuals are HIV/HCV co-infected [4, 5]. Interferon-based therapies for HCV have exhibited poor efficacy and significant toxicity in this population, limiting the capacity for large scale treatment delivery and success. However, current evidence indicates that direct acting antivirals (DAAs) exhibit excellent safety and efficacy in HIV-positive and HIV-negative individuals [6]. Moreover, the first DAAs became available under government subsidy to all HCV infected Australian adults in March 2016 [7].

Australia has one of the highest rates of HCV screening globally with an estimated 80% of the HCV infected population having been tested and diagnosed [5]. While current guidelines recommend HCV antibody testing for all HIV-positive people at initial presentation and at least annually thereafter (with a follow up HCV RNA test to confirm chronic infection if antibody testing is positive) [8], true rates of testing and incident infection in this group remain uncertain.

To allow monitoring of changes in HCV incidence after the introduction of DAA therapies it is important to evaluate past incidence trends. This will also facilitate the development of mathematical models to investigate the likely impact of DAA treatment scale-up and identify factors that could reduce the anticipated benefit of DAAs. Such work could have substantial public health implications as it is anticipated that appropriate use of DAAs in the HIV-positive population will lead to eventual elimination of HIV/HCV co-infection [9].

This paper assesses trends in HCV testing and re-testing among people attending Australian sexual health services, and evaluates trends in incident HCV infection among HIV-positive individuals. It also explores factors associated with HCV re-testing and incident infection among HIV-positive individuals.

## Methods

### Study population

The study population consisted of patients attending sexual health services participating in the Australian Collaboration for Coordinated Enhanced Sentinel Surveillance (ACCESS) project. ACCESS is a HIV/sexually transmitted infection sentinel surveillance network of sexual health services, general practice clinics, infectious diseases hospital outpatient clinics, and pathology laboratories. The methods behind this collaboration have been described in detail elsewhere [10]. For this analysis, de-identified demographic, behavioral, and clinical data were extracted from the patient management systems of 42 sexual health services using customized extraction scripts. Services were located in New South Wales, Victoria, Western Australia, Queensland, and the Northern Territory.

The ACCESS sexual health services network is broadly representative of sexual health services across the country. In Australia, sexual health services frequently manage the care of men who have sex with men (MSM) and people living with HIV. Nevertheless, a recent study of gay and bisexual men attending ACCESS general practice clinics found that about half were tested for a sexually transmitted infection between 2011 and 2015 [5]. The current analysis precedes a wider investigation of HCV testing and incidence across both sexual health services and general practice clinics which is currently being planned.

### Data analysis

Data on patient demographics, HIV and HCV testing were analyzed for patients who attended an ACCESS sexual health service between 2007 and 2015. Three main analyses were undertaken: (i) trends in HCV testing; (ii) trends in, and factors associated with, HCV re-testing; and (iii) trends in, and factors associated with, incident HCV infection. HCV testing/re-testing could consist of HCV antibody or RNA testing, and HCV positivity could include a positive HCV antibody or RNA test. Incident HCV infection was defined as a negative antibody test followed by either a positive antibody test or a positive RNA test, or a negative RNA test followed by a positive RNA test. Factors associated with HCV testing were not evaluated because of the bias introduced by high-risk individuals knowing their HCV status before attending an ACCESS sexual health service and therefore not undergoing testing.

HCV testing: Patients were included in the HCV testing rate population if they had at least one clinic visit after their first visit and were not known to be HCV-positive. Baseline was defined as the date of the first clinic visit. Annual rates of HCV testing were stratified by HIV status (HIV-negative individuals were retained as a comparison group). Follow up was censored at the time of testing HCV-positive, at HIV seroconversion, or 3 months after the last clinic visit. Those that were censored because of HIV seroconversion could re-enter the analysis as long as they had documentation of at least one further clinic visit after their date of HIV seroconversion.

HCV re-testing: Patients were only included in the HCV re-testing rate population if they had documentation of a negative HCV test and at least one further clinic visit. Baseline was defined as the date the first negative HCV test was undertaken. Annual rates of re-testing were stratified by HIV status. Censoring was as per the testing rate analysis. Those that were censored because of HIV seroconversion could re-enter the analysis as long as they had documentation of a negative HCV test while HIV-positive and at least one further clinic visit.

HCV incidence: HIV-positive individuals in our re-testing rate analysis were included in our HCV incidence population if they had at least one HCV test after a negative HCV antibody test, or at least one HCV RNA test after a negative HCV RNA test. The date of HCV infection was taken as the mid-point between the last negative HCV test and the first positive HCV test. Follow up was censored 3 months after the last negative HCV test for those that never returned a positive result.

Statistics: Poisson regression was used to assess the significance of time trends in HCV testing, re-testing and incidence, and to evaluate factors associated with HCV re-testing and incident HCV infection. Our analysis of factors associated with HCV re-testing was split by calendar year (between 2007 and 2011 and between 2012 and 2015). Baseline age, sex, country of birth, place of residence, sexual orientation, history of sex work (at any stage of study period), history of injecting drug use (at any stage of study period), and Aboriginal and Torres Strait Islander status were considered fixed covariates. CD4 cell count, HIV viral load, antiretroviral therapy use and calendar year were evaluated as time-updated covariates. Undetectable HIV viral load was defined as <200 copies/ml. Covariates were considered for inclusion in our multivariate models if they exhibited a univariate *p*-value <0.2. They were retained in the multivariate models if they exhibited an adjusted *p*-value <0.05. Patients with missing data for any of the covariates analyzed were included in all analyses, but incidence rate ratios (IRRs) for missing covariate categories are not reported. Stata (StataCorp, College Station, TX) version 14.1 was used for all statistical analysis.

Sensitivity analyses: We chose to include HCV RNA in our primary definitions of HCV testing/re-testing and incident HCV infection to account for the fact that HCV testing is often not performed as recommended [11]. Others have defined incident infection as seroconversion only [12, 13]. To enhance the comparability of our findings we repeated our main analyses using only antibody testing/re-testing and seroconversion as our definitions of HCV testing/re-testing and HCV positivity, respectively.

## Results

**Fig. 1:**
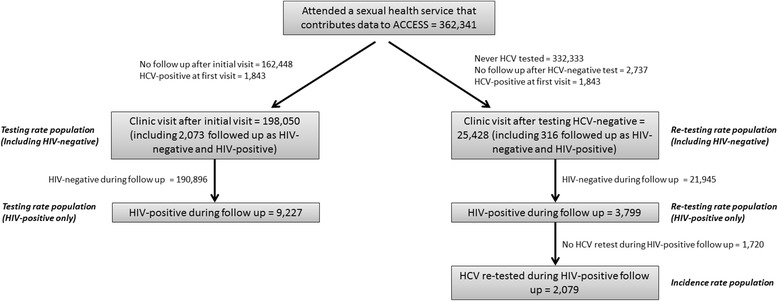
Study population selection process. ACCESS, Australian Collaboration for Coordinated Enhanced Sentinel Surveillance; HCV, hepatitis C virus; HIV, human immunodeficiency virus

Of 362,341 patients who attended a sexual health service contributing data to ACCESS, there were 198,050 who contributed follow up time in our testing rate analysis. However, since 2073 patients contributed data to both our HIV-positive and HIV-negative study populations (i.e., they seroconverted during follow up), the total number of HIV-negative and HIV-positive individuals contributing to each group was 190,896 and 9227, respectively. In our re-testing rate analysis, 25,248 patients contributed follow up – 21,945 HIV-negative and 3799 HIV-positive. Of the 3799 HIV-positive patients in our re-testing rate analysis, 2079 were re-tested at least once for HCV after their initial negative test and were therefore included in our incidence analysis. Figure 1 provides a comprehensive breakdown of the patient selection process.

### HCV testing

Baseline characteristics of the HCV testing rate population (HIV-positive and HIV-negative) are presented in Additional file 1: Table S1. In the HIV-positive group, median age was 40 years (interquartile range [IQR] 31–48) and 85.5% were male. In the HIV-negative group, median age was 27 years [IQR 23–36] and 58.0% were male.

**Fig. 2:**
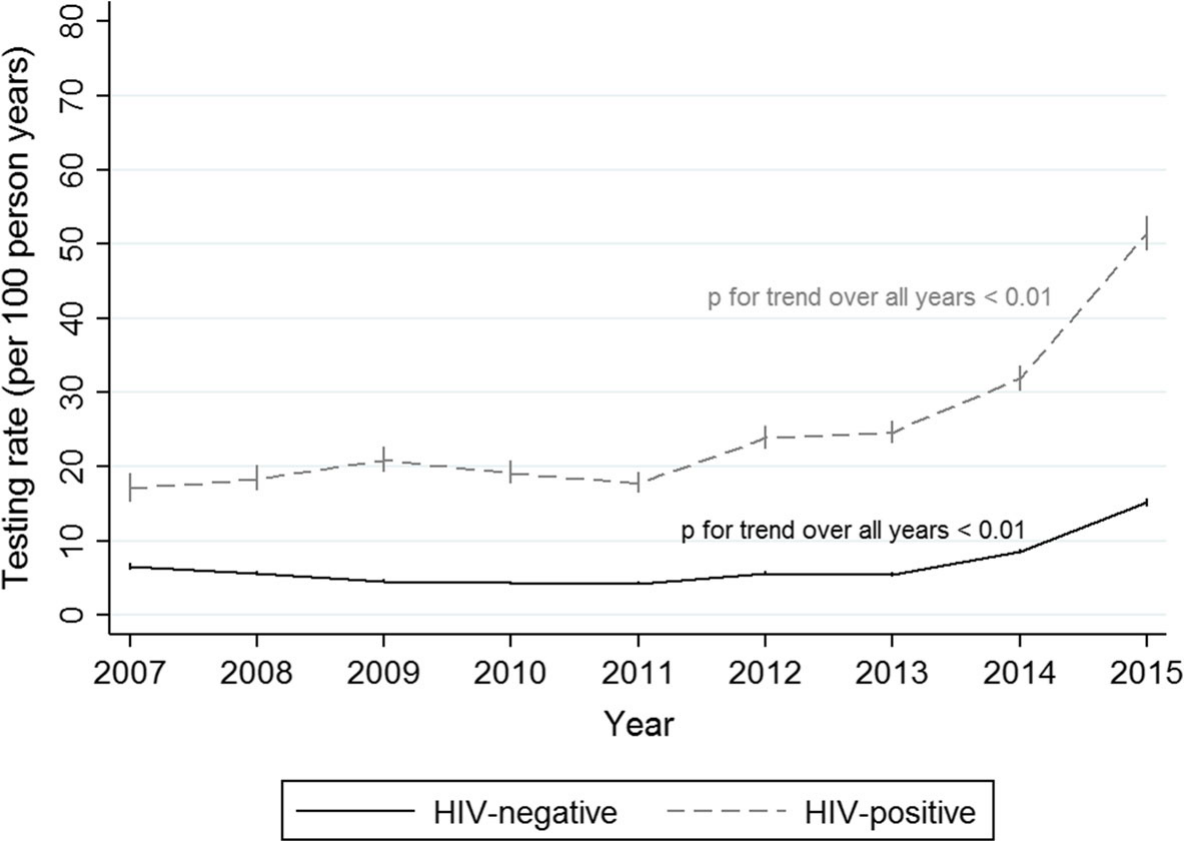
Rates of HCV testing in HIV-positive (*n* = 9227) and HIV-negative (*n* = 190,896) people. There were 2073 individuals who HIV seroconverted during follow up and contributed person years to both HIV-positive and HIV-negative study populations. Error bars represent 95% confidence interval. HCV, hepatitis C virus; HIV, human immunodeficiency virus

Figure 2 shows that, among HIV-positive individuals, the rate of HCV testing increased from 17.1 tests per 100 person years (95% confidence interval [CI] 15.2–19.1) in 2007 to 51.4 tests per 100 person years (95% CI 49.2–53.8) in 2015 (*p* for trend <0.01). In HIV-negative individuals, the rate increased from 6.5 tests per 100 person years (95% CI 6.1–6.9) in 2007 to 15.2 tests per 100 person years (95%CI 14.6–15.8) in 2015 (*p* for trend <0.01). A steady increase in HCV testing began after 2011 in both groups.

Similar trends were found in our sensitivity analyses focusing on HCV antibody testing. In HIV-positive individuals, the rate of HCV antibody testing increased from 7.9 tests per 100 person years (95% CI 6.7–9.3) to 39.4 tests per 100 person years (95%CI 37.4–41.4) between 2007 and 2015 (*p* for trend <0.01). Among HIV-negative individuals over the same period, the rate increased from 2.3 tests per 100 person years (95% CI 2.1–2.6) to 11.1 tests per 100 person years (95% CI 10.6–11.6; *p* for trend <0.01).

### HCV re-testing

Baseline characteristics of the HCV re-testing rate population (HIV-positive and HIV-negative) are presented in Additional file 1: Table S2. Among the HIV-positive group, median age was 39 years (IQR 30–48) and 88.2% were male. In the HIV-negative group, median age was 29 years [IQR 23–38] and 61.7% were male.

**Fig. 3:**
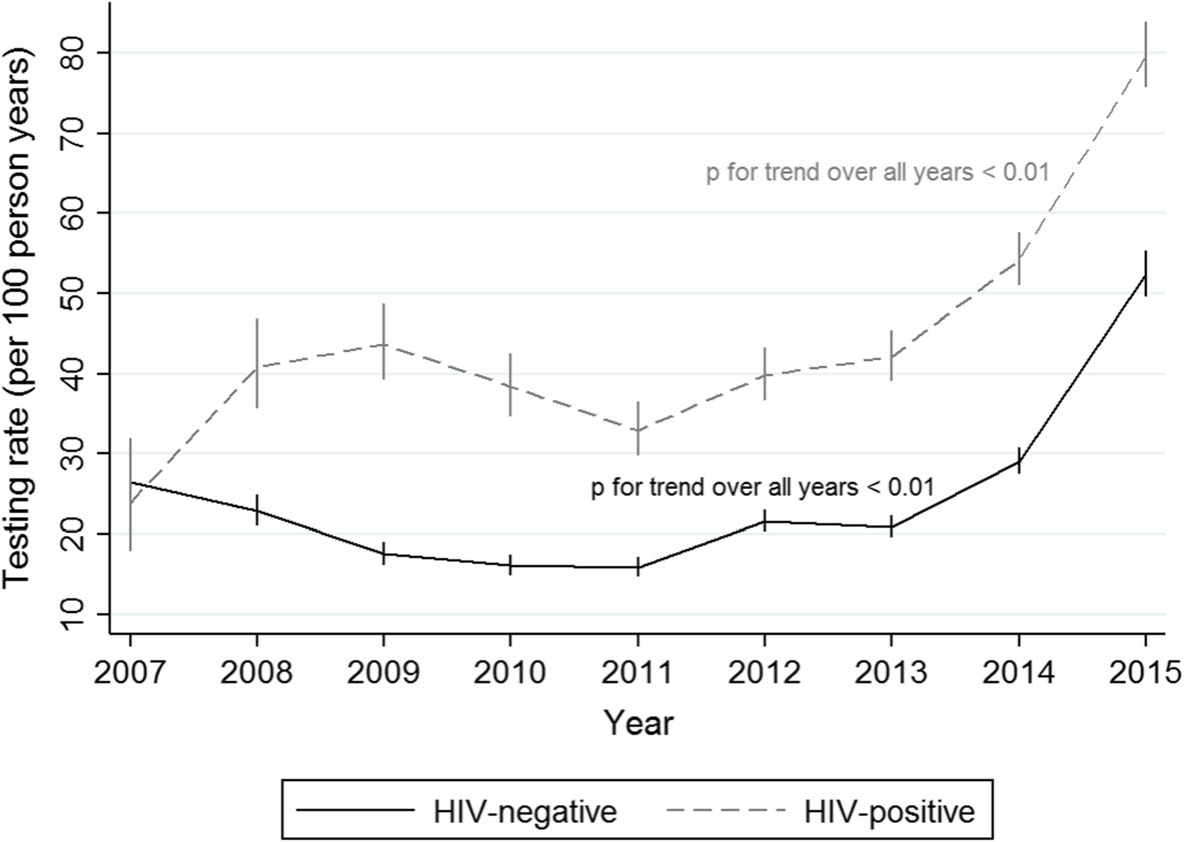
Rates of HCV re-testing in HIV-positive (*n* = 3799) and HIV-negative (*n* = 21,945) people. There were 316 individuals who HIV seroconverted during follow up and contributed person years to both HIV-positive and HIV-negative study populations. Error bars represent 95% confidence interval. HCV, hepatitis C virus; HIV, human immunodeficiency virus

In HIV-positive individuals, the rate of HCV re-testing increased from 23.9 tests per 100 person years (95% CI 17.9–32.0) in 2007 to 79.7 tests per 100 person years (95% CI 75.7–83.8) in 2015 (*p* for trend <0.01). Among HIV-negative individuals, the rate increased from 26.5 tests per 100 person years (95% CI 23.5–29.8) in 2007 to 52.4 tests per 100 person years (95% CI 49.6–55.3) in 2015 (*p* for trend <0.01). An increase in HCV re-testing began after 2011 (see Fig. 3).

Table 1 shows that, among HIV-positive individuals, a history of injecting drug use and identifying as MSM were the only factors significantly associated with a shorter time to HCV re-testing between both 2007–2011 and 2012–2015. Country of birth other than Australia, residing in a regional/remote location, and identifying as Aboriginal or Torres Strait Islander were associated with a greater rate of HCV re-testing between 2007 and 2011 and younger age, residing in a major city, and later calendar year were associated with a greater rate of HCV re-testing between 2012 and 2015.

**Table 1:** Factors associated with HCV re-testing in HIV-positive people (*n* = 3799)

	Between 2007 and 2011							Between 2012 and 2015						
	HCV re-tested	Person years	Rate per 100 person years (95% CI)	Univariate IRR (95% CI)	*p*	Multivariate IRR (95% CI)	*p*	HCV re-tested	Person years	Rate per 100 person years (95% CI)	Univariate IRR (95% CI)	*p*	Multivariate IRR (95% CI)	*p*
Age														
Every additional 5 years	666	2001.5	33.3 (30.8–35.9)	1.0 (0.9–1.0)	0.07			1323	2862.2	46.2 (43.8–48.8)	0.9 (0.9–1.0)	<0.01	1.0 (0.9–1.0)*	<0.01
Sex														
Male	606	1762.8	34.4 (31.7–37.2)	1.0				1195	2456.3	48.6 (46.0–51.5)	1.0			
Female	60	238.8	25.1 (19.5–32.4)	0.7 (0.6–1.0)	0.02			128	405.9	31.5 (26.5–37.5)	0.6 (0.5–0.8)	<0.01		
Country of birth														
Australia	449	1397.4	32.1 (29.3–35.2)	1.0		1.0		871	1903.0	45.8 (42.8–48.9)	1.0			
Other	217	604.1	35.9 (31.4–41.0)	1.1 (1.0–1.3)	0.18	1.3 (1.1–1.6)	<0.01	452	959.2	47.1 (43.0–51.7)	1.0 (0.9–1.2)	0.62		
Place of residence														
Major city	339	1092.4	31.0 (27.9–34.5)	1.0		1.0		944	1726.4	54.7 (51.3–58.3)	1.0		1.0	
Regional/remote	316	878.1	36.0 (32.2–40.2)	1.2 (1.0–1.4)	0.06	1.3 (1.1–1.5)	<0.01	367	1085.6	33.8 (30.5–37.4)	0.6 (0.5–0.7)	<0.01	0.7 (0.6–0.8)	<0.01
Unknown	11	31.0	35.5 (19.7–64.1)	–		–		12	50.2	23.9 (13.6–42.1)	–		–	
Sexual orientation														
MSM	528	1416.0	37.3 (34.2–40.6)	1.7 (1.3–2.1)	0.00	1.8 (1.4–2.3)	<0.01	996	1941.6	51.3 (48.2–54.6)	1.3 (1.1–1.5)	<0.01	1.2 (1.0–1.4)	0.03
Heterosexual male	78	346.7	22.5 (18.0–28.1)	1.0		1.0		198	512.7	38.6 (33.6–44.4)	1.0		1.0	
Unknown	60	238.8	25.1 (19.5–32.4)	–		–		129	407.9	31.6 (26.6–37.6)	–		–	
Ever performed sex work														
No	649	1942.7	33.4 (30.9–36.1)	1.0				1278	2783.7	45.9 (43.5–48.5)	1.0			
Yes	17	58.8	28.9 (18.0–46.5)	0.9 (0.5–1.4)	0.56			45	78.5	57.3 (42.8–76.8)	1.2 (0.9–1.7)	0.14		
Ever injected drugs														
No	507	1649.6	30.7 (28.2–33.5)	1.0		1.0		1126	2513.1	44.8 (42.3–47.5)	1.0		1.0	
Yes	109	223.9	48.7 (40.3–58.7)	1.6 (1.3–1.9)	<0.01	1.7 (1.4–2.1)	<0.01	148	256.6	57.7 (49.1–67.8)	1.3 (1.1–1.5)	<0.01	1.3 (1.1–1.5)	0.01
Unknown	50	128.0	39.1 (29.6–51.5)	–		–		49	92.5	52.9 (40.0–70.1)	–		–	
Aboriginal or Torres Strait Islander														
No	629	1915.1	32.8 (30.4–35.5)	1.0		1.0		1270	2748.6	46.2 (43.7–48.8)	1.0			
Yes	37	86.4	42.8 (31.0–59.1)	1.3 (0.9–1.8)	0.12	1.4 (1.0–2.0)	0.04	53	113.6	46.6 (35.6–61.1)	1.0 (0.8–1.3)	0.95		
Current CD4 cell count (cells/mm^3^)														
≥ 500	164	596.3	27.5 (23.6–32.1)	1.0				501	1107.2	45.2 (41.5–49.4)	1.0			
< 500	158	567.2	27.9 (23.8–32.6)	1.0 (0.8–1.3)	0.91			340	786.7	43.2 (38.9–48.1)	1.0 (0.8–1.1)	0.51		
Unknown	344	838.1	41.0 (36.9–45.6)	–				482	968.3	49.8 (45.5–54.4)	–			
Current HIV RNA														
Undetectable	299	898.5	33.3 (29.7–37.3)	1.0				758	1585.1	47.8 (44.5–51.3)	1.0			
Detectable	167	536.3	31.1 (26.8–36.2)	0.9 (0.8–1.1)	0.49			243	465.2	52.2 (46.1–59.2)	1.1 (0.9–1.3)	0.23		
Unknown	200	566.7	35.3 (30.7–40.5)	–				322	811.9	39.7 (35.6–44.2)	–			
Current ART use														
Yes	366	1121.3	32.6 (29.5–36.2)	1.0				919	1851.9	49.6 (46.5–52.9)	1.0			
No	100	313.5	31.9 (26.2–38.8)	1.0 (0.8–1.2)	0.84			82	198.5	41.3 (33.3–51.3)	0.8 (0.7–1.0)	0.11		
Unknown	200	566.7	35.3 (30.7–40.5)	–				322	811.9	39.7 (35.6–44.2)	–			
Current calendar year~														
2007 / 2012	74	236.7	31.3 (24.9–39.3)	1.0		1.0		279	760.9	36.7 (32.6–41.2)	1.0		1.0	
2008 / 2013	167	424.3	39.4 (33.8–45.8)	1.3 (1.0–1.7)	0.10	1.3 (1.0–1.7)	0.07	361	819.4	44.1 (39.7–48.8)	1.2 (1.0–1.4)	0.02	1.2 (1.0–1.4)	0.02
2009 / 2014	155	487.2	31.8 (27.2–37.2)	1.0 (0.8–1.3)	0.90	1.1 (0.8–1.4)	0.66	414	808.1	51.2 (46.5–56.4)	1.4 (1.2–1.6)	<0.01	1.4 (1.2–1.6)	<0.01
2010 / 2015	158	487.3	32.4 (27.7–37.9)	1.0 (0.8–1.4)	0.80	1.1 (0.8–1.4)	0.52	269	473.8	56.8 (50.4–64.0)	1.5 (1.3–1.8)	<0.01	1.5 (1.3–1.8)	<0.01
2011 / -	112	366.0	30.6 (25.4–36.8)	1.0 (0.7–1.3)	0.89	1.1 (0.8–1.4)	0.74	–	–	–	–		–	

Missing categories were included in models but coefficients are not shown. *IRR (95%CI) to two decimal places was 0.96 (0.94–0.98); ~Calendar year for 2007–2011 model / calendar year for 2012–2015 model. Despite calendar year not reaching significance in the multivariate 2007–2011 model, it was retained to allow comparison with the unadjusted testing rates in Fig. 2; HCV, hepatitis C virus; HIV, human immunodeficiency virus; CI, confidence interval; IRR, incidence rate ratio; MSM, men who have sex with men; ART, antiretroviral therapy

Similar results were found in our sensitivity analyses. In HIV-positive individuals, the rate of HCV re-testing increased from 21.2 tests per 100 person years (95% CI 13.3–33.6) to 76.9 tests per 100 person years (95% CI 72.2–81.8) between 2007 and 2015 (*p* for trend <0.01). Among HIV-negative individuals over the same period, the rate increased from 17.9 tests per 100 person years (95% CI 13.9–23.2) to 49.3 tests per 100 person years (95% CI 45.9–52.9; *p* for trend <0.01). A history of injecting drug use and identifying as MSM remained important factors associated with HCV re-testing between both 2007–2011 (IRR 2.1, 95% CI 1.6–2.9, *p* < 0.01 for history of injecting versus no history and IRR 1.8, 95% CI 1.2–2.9, *p* = 0.01 for MSM versus heterosexual male) and 2012–2015 (IRR 1.2, 95% CI 1.0–1.5, *p* = 0.09 for history of injecting versus no history and IRR 1.3, 95% CI 1.1–1.6, *p* = 0.01 for MSM versus heterosexual male). Those residing in a major city were also subject to a higher rate of re-testing between 2012 and 2015 (IRR 0.7, 95% CI 0.6–0.8, *p* < 0.01 for regional/remote versus major city).

### HCV incidence

Characteristics of the HCV incidence rate population at the time of their first negative HCV test are presented in Additional file 1: Table S3. The median age was 41 years (IQR 32–50) and 90.7% were male. Median CD4 cell count was 510 cells/mm^3^ (IQR 356–672), 74.9% of patients with a known treatment status were using antiretroviral therapy, and 50.2% of patients with a documented HIV viral load had a level that was undetectable.

Over the study period, 157 incident HCV infections occurred at a rate of 2.5 events per 100 person years (95% CI 2.1–2.9). Between 2007 and 2009, 2010–2011, 2012–2013 and 2014–2015, rates of incident HCV were 0.8 (95% CI 0.4–1.6), 1.5 (95% CI 1.0–2.3), 3.9 (95% CI 3.1–4.9) and 2.7 (95% CI 2.0–3.5) events per 100 person years, respectively (*p* for trend <0.01). During the same periods, rates among those who identified as MSM without a history of injecting drug use were 0.4 (95% CI 0.1–1.4), 1.2 (95% CI 0.6–2.1), 3.0 (95% CI 2.2–4.1) and 2.5 (95% CI 1.8–3.5) events per 100 person years, respectively (*p* for trend <0.01). Among MSM with a history of injecting drug use, rates were 1.8 (95% CI 0.5–7.4), 2.6 (95% CI 1.0–6.7), 10.7 (95% CI 6.9–16.3) and 2.7 (95% CI 1.1–6.6) events per 100 person years, respectively (*p* for trend = 0.24). In our final model adjusted for calendar year, incident HCV infection was associated with younger age, living in a major city, being a heterosexual male, having a history of injecting drug use, and being an Aboriginal or Torres Strait Islander (Table 2). When sexual orientation and injecting drug use were included as an interaction term, there was no difference in the rate of incident HCV infection between non-injecting MSM and non-injecting male heterosexuals (IRR 1.4 with non-injecting MSM as the reference group, 95% CI 0.8–2.4, *p* = 0.25), but a substantial difference between injecting MSM and injecting male heterosexuals (IRR 3.1 with injecting MSM as the reference group, 95% CI 1.5–6.1, *p* < 0.01).

**Table 2:** Factors associated with incident HCV infection in HIV-positive people (*n* = 2079)

	Incident infections	Person years	Rate per 100 person years (95% CI)	Univariate IRR (95% CI)	*p*	Multivariate IRR (95% CI)	*p*
Age							
Every additional 5 years	157	6279.7	2.5 (2.1–2.9)	0.8 (0.8–0.9)	<0.01	0.9 (0.8–0.9)	<0.01
Sex							
Male	141	5772.2	2.4 (2.1–2.9)	1.0			
Female	16	507.5	3.2 (1.9–5.1)	1.3 (0.8–2.2)	0.33		
Country of birth							
Australia	99	4316.1	2.3 (1.9–2.8)	1.0			
Other	58	1963.6	3.0 (2.3–3.8)	1.3 (0.9–1.8)	0.13		
Place of residence							
Major city	132	3795.0	3.5 (2.9–4.1)	1.0		1.0	
Regional/remote	25	2414.6	1.0 (0.7–1.5)	0.3 (0.2–0.5)	<0.01	0.3 (0.2–0.5)	<0.01
Unknown	0	70.1	–	–		–	
Sexual orientation							
MSM	115	4950.0	2.3 (1.9–2.8)	0.7 (0.5–1.1)	0.15	0.5 (0.4–0.8)	0.01
Heterosexual male	26	820.5	3.2 (2.2–4.7)	1.0		1.0	
Unknown	16	509.1	3.1 (1.9–5.1)	–		–	
Ever performed sex work							
No	151	6086.7	2.5 (2.1–2.9)	1.0			
Yes	6	192.9	3.1 (1.4–6.9)	1.3 (0.6–2.8)	0.59		
Ever injected drugs							
No	111	5249.1	2.1 (1.8–2.5)	1.0		1.0	
Yes	46	795.6	5.8 (4.3–7.7)	2.7 (1.9–3.9)	<0.01	2.5 (1.8–3.5)	<0.01
Unknown	0	234.9	–	–		–	
Aboriginal or Torres Strait Islander							
No	146	5992.9	2.4 (2.1–2.9)	1.0		1.0	
Yes	11	286.8	3.8 (2.1–6.9)	1.6 (0.9–2.9)	0.15	2.0 (1.1–3.7)	0.03
Current CD4 cell count (cells/mm^3^)							
≥ 500	34	1648.2	2.1 (1.5–2.9)	1.0			
< 500	17	1116.1	1.5 (0.9–2.5)	0.7 (0.4–1.3)	0.31		
Unknown	106	3515.4	3.0 (2.5–3.6)	–			
Current HIV RNA							
Undetectable	63	2647.6	2.4 (1.9–3.0)	1.0			
Detectable	21	869.0	2.4 (1.6–3.7)	1.0 (0.6–1.7)	0.95		
Unknown	73	2763.1	2.6 (2.1–3.3)	–			
Current ART use							
Yes	74	3008.6	2.5 (2.0–3.1)	1.0			
No	10	508.0	2.0 (1.1–3.7)	0.8 (0.4–1.5)	0.51		
Unknown	73	2763.1	2.6 (2.1–3.3)	–			
Current time period							
2007–09	8	1027.6	0.8 (0.4–1.6)	1.0		1.0	
2010–11	21	1385.5	1.5 (1.0–2.3)	1.8 (0.8–4.1)	0.15	1.7 (0.8–3.9)	0.19
2012–13	77	1960.5	3.9 (3.1–4.9)	4.5 (2.2–9.4)	<0.01	4.1 (2.0–8.5)	<0.01
2014–15	51	1906.0	2.7 (2.0–3.5)	2.6 (1.2–5.4)	0.01	2.2 (1.1–4.7)	0.04

Missing categories were included in models but coefficients are not shown. HCV, hepatitis C virus; HIV, human immunodeficiency virus; CI, confidence interval; IRR, incidence rate ratio; MSM, men who have sex with men; ART, antiretroviral therapy

In our sensitivity analysis focusing on HCV antibody testing, 14 incident HCV infections occurred at a rate of 0.4 events per 100 person years (95% CI 0.2–0.6). Between 2007 and 2009, 2010–2011, 2012–2013 and 2014–2015, rates of incident HCV were 0.2 (95% CI 0.0–1.6), 0.1 (95% CI 0.0–1.0), 0.6 (95%CI 0.3–1.2) and 0.3 (95% CI 0.1–0.8) events per 100 person years, respectively (*p* for trend = 0.59). The low number of seroconversions observed precluded us from developing a meaningful multivariate model, however, a history of injecting drug use was strongly associated with incident HCV infection in our univariate model (IRR 8.4, 95% CI 2.9–24.2, *p* < 0.01 versus no history).

## Discussion

HCV testing and re-testing in Australian sexual health services has increased substantially among HIV-positive (and HIV-negative) people since 2011. Rates of re-testing were consistently high among MSM and injecting drug users between 2007 and 2015, however, there was a marked increase in testing rates during the latter years of the study period among those residing in a major city. The rate of incident HCV infection increased between 2007 and 2013 but remained stable between 2014 and 2015. Incident HCV was associated with a history of injecting drug use, particularly among heterosexual males.

HIV-positive MSM have an increased risk of HCV infection compared with HIV-negative MSM, possibly due to higher rates of high-risk sexual behavior [14]. Current Australian guidelines recommend routine HCV testing among HIV-positive MSM [15]. This explains why HCV testing and re-testing rates were consistently higher over time among our HIV-positive group. The increasing rates of HCV testing and re-testing observed are probably linked to a growing awareness of sexual HCV transmission [16] and, more recently, increasing awareness of DAA availability [17]. The number of state and national campaigns aiming to increase HCV and DAA awareness has increased substantially over the past decade [18, 19]. Moreover, national advisory groups such as the Australasian Society for HIV, Viral Hepatitis and Sexual Health Medicine have actively sought to keep prescribers informed about DAA availability [20]. Given that non-sterile injecting practices and sexual behaviors that increase the potential for exposure to blood increase the possibility of transmitting HCV, it was unsurprising to find that injecting drug users and MSM were subject to high rates of HCV re-testing. Of note, however, the increase in HCV re-testing rates between the periods 2007–2011 and 2012–2015 among patients living in a major city was contrasted by a small decline in testing among those living in regional/remote locations. This suggests that knowledge of DAA availability may be lacking outside of Australia’s main urban centers.

Other recent studies from Australia and abroad have described an overall HCV incidence rate in HIV-positive people similar to that reported here (2.5 events per 100 person years) [12, 13, 21–27]. In contrast to our findings, however, which show HCV incidence increased between 2007 and 2013 then plateaued, data from the Swiss HIV Cohort suggests HCV incidence has increased consistently since 2000 among MSM [13] and results from the UK Collaborative HIV Cohort indicate that HCV incidence remained stable between 2004 and 2011 among MSM [12]. There are two important methodological differences between these two studies and ours. Firstly, unlike our study, patients enrolled in the Swiss and UK cohorts underwent routine HCV testing. The low rates of testing/re-testing during the earlier years of our study period could have caused us to underestimate HCV incidence. However, given HCV diagnoses were uncommon and that all patients in our incidence analysis were tested at least twice for HCV, it is unlikely that testing rates substantially influenced our results. The other main difference is that the Swiss and UK studies defined incident infection as HCV seroconversion. Unfortunately, a preliminary assessment of our data indicated that, consistent with findings from the US [11], HCV testing was often not performed as recommended. This meant that the group of patients we identified as having experienced an incident HCV infection included an unknown number of re-infected patients. Nevertheless, our main and sensitivity analyses showed similar trends in both HCV testing and incident infection rates.

Interestingly, another Australian study recently found no change in HCV incidence between 2008 and 2016 among MSM [21]. Of note, this single-center study specifically asked HCV cases about injecting drug use and excluded those reporting a positive history of injecting regardless of what was noted in their records. Our analysis relied upon routinely collected surveillance data in which injecting drug use was probably under-reported. This is supported by the results of our incidence model. While those with a history of injecting drug use had a high risk of incident HCV infection, it is likely that the other significantly associated factors (younger age, living in a major city, being a heterosexual male, and being Aboriginal and Torres Strait Islander) were largely the result of residual confounding. Each of these additional factors are known to be associated with injecting drug use [28, 29]. This implies that the increase in HCV incidence among Australia’s HIV-positive population may have been driven by injecting drug use and that sexual transmission has not changed substantially. This could be related to increased rates of unsafe injecting among sub-populations of the HIV-positive community. For example, between 2011 and 2014, the proportion of HIV infections attributable to IDU increased dramatically among Aboriginal and Torres Strait Islanders [5]. Importantly, heterosexual males that inject drugs appear to be at especially high risk of contracting HCV. This finding warrants further investigation.

There are several limitations to bear in mind when evaluating the results of this work. Firstly, we were unable to link records between the sexual health services involved. Therefore, patients could have re-entered the analysis at multiple services. Additionally, we had limited information on sexual risk behavior and there is a high likelihood that injecting drug use was under-reported. Our analysis populations represent only a sample of the approximately 21,000 HIV patients who are currently diagnosed and in care in Australia [5], which means our rate estimates reflect only a subset of the total number of tests and diagnoses carried out nationwide. As mentioned earlier, an analysis of HCV testing and incidence across both sexual health services and general practice clinics involved in ACCESS is currently being planned. We were not able to conduct an appropriate analysis of factors associated with HCV testing because of the bias introduced by high-risk individuals knowing their HCV status prior to enrolment and therefore not undergoing testing. Further details on HCV testing rates and factors associated with HCV testing should form the topic of future investigation. Finally, while our testing and re-testing rate sensitivity analyses supported our main findings, our incidence rate sensitivity analyses were impeded by a very low number of outcomes.

## Conclusions

The increasing rates of HCV testing and re-testing reported here are encouraging. High rates of HCV testing and re-testing among high-risk groups in Australia will assist strategies to achieve HCV elimination through rapid treatment scale up. Continued monitoring of HCV incidence in the HIV-positive population, particularly those with a history of injecting drug use, is essential for guiding both HCV prevention and treatment strategies.

## Additional file

Additional file 1: Table S1. Characteristics of the HIV-positive and HIV-negative HCV testing rate populations at the time of their first clinic visit. Values are presented as n (% of patients) unless otherwise indicated. *2073 individuals HIV seroconverted during follow up and contributed person years to both HIV-positive and HIV-negative study populations; HIV, human immunodeficiency virus; HCV, hepatitis C virus; IQR, interquartile range; MSM, men who have sex with men. **Table S2.** Characteristics of the HIV-positive and HIV-negative HCV re-testing rate populations at the time of their first documented HCV test. Values are presented as n (% of patients) unless otherwise indicated. *316 individuals HIV seroconverted during follow up and contributed person years to both HIV-positive and HIV-negative study populations; HIV, human immunodeficiency virus; HCV, hepatitis C virus; IQR, interquartile range; MSM, men who have sex with men. **Table S3.** Characteristics of the HCV incidence population at the time of their first negative HCV test. Values are presented as n (% of patients) unless otherwise indicated. HCV, hepatitis C virus; IQR, interquartile range; MSM, men who have sex with men; HIV, human immunodeficiency virus; ART, antiretroviral therapy. (DOCX 34 kb)

## Abbreviations

ACCESS: Australian Collaboration for Coordinated Enhanced Sentinel Surveillance
CI: Confidence interval
DAA: Direct acting antiviral
HCV: Hepatitis C Virus
HIV: Human Immunodeficiency Virus
IQR: Interquartile range
IRR: Incidence rate ratio
MSM: Men who have sex with men
