# Supplementary material for: Hepatitis C testing and re-testing among people attending sexual health services in Australia, and hepatitis C incidence among people with human immunodeficiency virus: analysis of national sentinel surveillance data
Source: BMC Infect Dis. 2017 Dec 1;17:740. doi: 10.1186/s12879-017-2848-0 (PMC5709850; doi:10.1186/s12879-017-2848-0)
Supplement: Additional file 1: Table S1. — Characteristics of the HIV-positive and HIV-negative HCV testing rate populations at the time of their first clinic visit. Values are presented as n (% of patients) unless otherwise indicated. *2073 individuals HIV seroconverted during follow up and contributed person years to both HIV-positive and HIV-negative study populations; HIV, human immunodeficiency virus; HCV, hepatitis C virus; IQR, interquartile range; MSM, men who have sex with men. Table S2. Characteristics of the HIV-positive and HIV-negative HCV re-testing rate populations at the time of their first documented HCV test. Values are presented as n (% of patients) unless otherwise indicated. *316 individuals HIV seroconverted during follow up and contributed person years to both HIV-positive and HIV-negative study populations; HIV, human immunodeficiency virus; HCV, hepatitis C virus; IQR, interquartile range; MSM, men who have sex with men. Table S3. Characteristics of the HCV incidence population at the time of their first negative HCV test. Values are presented as n (% of patients) unless otherwise indicated. HCV, hepatitis C virus; IQR, interquartile range; MSM, men who have sex with men; HIV, human immunodeficiency virus; ART, antiretroviral therapy. (DOCX 34 kb) [file 12879_2017_2848_MOESM1_ESM.docx]

**Table S1**

|  | **HIV-positive (n=9,227)*** | **HIV-negative (n=190,896)*** |
| --- | --- | --- |
| **Age (years)** |  |  |
| Median (IQR) | 40 (31-48) | 27 (23-36) |
| **Sex^** |  |  |
| Male | 7,891 (85.5) | 110,664 (58.0) |
| Female | 1,336 (14.5) | 80,198 (42.0) |
| Other | 0 (0.0) | 34 (0.0) |
| **Country of birth** |  |  |
| Australia | 5,812 (63.0) | 113,932 (59.7) |
| Other | 3,415 (37.0) | 76,964 (40.3) |
| **Place of residence** |  |  |
| Major city | 6,371 (69.1) | 130,617 (68.4) |
| Regional/remote | 2,634 (28.6) | 49,045 (25.7) |
| Unknown | 222 (2.4) | 11,234 (5.9) |
| **Sexual orientation** |  |  |
| MSM | 5,714 (61.9) | 42,140 (22.1) |
| Heterosexual male | 2,167 (23.5) | 68,300 (35.8) |
| Unknown | 1,346 (14.6) | 80,456 (42.2) |
| **Ever performed sex work** |  |  |
| No | 8,950 (97.0) | 175,743 (92.1) |
| Yes | 277 (3.0) | 15,153 (7.9) |
| **Ever injected drugs** |  |  |
| No | 5,749 (62.3) | 106,083 (55.6) |
| Yes | 889 (9.6) | 7,075 (3.7) |
| Unknown | 2,589 (28.1) | 77,738 (40.7) |
| **Aboriginal or Torres Strait Islander** |  |  |
| No | 8,912 (96.6) | 182,012 (95.4) |
| Yes | 315 (3.4) | 8,884 (4.7) |

**Table S2**

|  | **HIV-positive (n=3,799)*** | **HIV-negative (n=21,945)*** |
| --- | --- | --- |
| **Age (years)** |  |  |
| Median (IQR) | 39 (30-48) | 29 (23-38) |
| **Sex** |  |  |
| Male | 3,350 (88.2) | 13,530 (61.7) |
| Female | 448 (11.8) | 8,409 (38.3) |
| Other | 1 (0.0) | 6 (0.0) |
| **Country of birth** |  |  |
| Australia | 2,452 (64.5) | 16,606 (75.7) |
| Other | 1,347 (35.5) | 5,339 (24.3) |
| **Place of residence** |  |  |
| Major city | 2,409 (63.4) | 11,312 (51.6) |
| Regional/remote | 1,326 (34.9) | 10,237 (46.7) |
| Unknown | 64 (1.7) | 396 (1.8) |
| **Sexual orientation** |  |  |
| MSM | 2,720 (71.6) | 5,921 (27.0) |
| Heterosexual male | 629 (16.6) | 7,601 (34.6) |
| Unknown | 450 (11.9) | 8,423 (38.4) |
| **Ever performed sex work** |  |  |
| No | 3,668 (96.6) | 19,902 (90.7) |
| Yes | 131 (3.5) | 2,043 (9.3) |
| **Ever injected drugs** |  |  |
| No | 3,067 (80.7) | 17,828 (81.2) |
| Yes | 424 (11.2) | 2,611 (11.9) |
| Unknown | 308 (8.1) | 1,506 (6.9) |
| **Aboriginal or Torres Strait Islander** |  |  |
| No | 3,631 (95.6) | 19,455 (88.7) |
| Yes | 168 (4.4) | 2,490 (11.4) |

**Table S3**

|  | **All (n=2,079)** |
| --- | --- |
| **Age (years)** |  |
| Median (IQR) | 41 (32-50) |
| **Sex** |  |
| Male | 1,885 (90.7) |
| Female | 194 (9.3) |
| **Country of birth** |  |
| Australia | 1,378 (66.3) |
| Other | 701 (33.7) |
| **Place of residence** |  |
| Major city | 1,343 (64.6) |
| Regional/remote | 713 (34.3) |
| Unknown | 23 (1.1) |
| **Sexual orientation** |  |
| MSM | 1,595 (76.7) |
| Heterosexual male | 289 (13.9) |
| Unknown | 195 (9.4) |
| **Ever performed sex work** |  |
| No | 2,010 (96.7) |
| Yes | 69 (3.3) |
| **Ever injected drugs** |  |
| No | 1,701 (81.8) |
| Yes | 268 (12.9) |
| Unknown | 110 (5.3) |
| **Aboriginal or Torres Strait Islander** |  |
| No | 1,984 (95.4) |
| Yes | 95 (4.6) |
| **Current CD4 cell count (cells/mm^3^)** |  |
| Median (IQR) | 510 (356-672) |
| Unknown | 1,341 (64.5) |
| **Current HIV RNA** |  |
| Undetectable | 487 (23.4) |
| Detectable | 484 (23.3) |
| Unknown | 1,108 (53.3) |
| **Current ART use** |  |
| Yes | 727 (35.0) |
| No | 244 (11.7) |
| Unknown | 1,108 (53.3) |
